# Cerebrospinal Fluid Neurofilament Light Chain (NfL) Predicts Disease Aggressiveness in Amyotrophic Lateral Sclerosis: An Application of the D50 Disease Progression Model

**DOI:** 10.3389/fnins.2021.651651

**Published:** 2021-04-06

**Authors:** Marie Dreger, Robert Steinbach, Nayana Gaur, Klara Metzner, Beatrice Stubendorff, Otto W. Witte, Julian Grosskreutz

**Affiliations:** ^1^Hans Berger Department of Neurology, Jena University Hospital, Jena, Germany; ^2^Center for Healthy Ageing, Jena University Hospital, Jena, Germany

**Keywords:** amyotrophic lateral sclerosis, neurofilaments, NfL, cerebrospinal fluid, prognostic biomarker

## Abstract

Amyotrophic lateral sclerosis (ALS) is a relentlessly progressive neurodegenerative disorder. As previous therapeutic trials in ALS have been severely hampered by patients’ heterogeneity, the identification of biomarkers that reliably reflect disease progression represents a priority in ALS research. Here, we used the D50 disease progression model to investigate correlations between cerebrospinal fluid (CSF) neurofilament light chain (NfL) levels and disease aggressiveness. The D50 model quantifies individual disease trajectories for each ALS patient. The value D50 provides a unified measure of a patient’s overall disease aggressiveness (defined as time taken in months to lose 50% of functionality). The relative D50 (rD50) reflects the individual disease covered and can be calculated for any time point in the disease course. We analyzed clinical data from a well-defined cohort of 156 patients with ALS. The concentration of NfL in CSF samples was measured at two different laboratories using the same procedure. Based on patients’ individual D50 values, we defined subgroups with high (<20), intermediate (20–40), or low (>40) disease aggressiveness. NfL levels were compared between these subgroups via analysis of covariance, using an array of confounding factors: age, gender, clinical phenotype, frontotemporal dementia, rD50-derived disease phase, and analyzing laboratory. We found highly significant differences in NfL concentrations between all three D50 subgroups (*p* < 0.001), representing an increase of NfL levels with increasing disease aggressiveness. The conducted analysis of covariance showed that this correlation was independent of gender, disease phenotype, and phase; however, age, analyzing laboratory, and dementia significantly influenced NfL concentration. We could show that CSF NfL is independent of patients’ disease covered at the time of sampling. The present study provides strong evidence for the potential of NfL to reflect disease aggressiveness in ALS and in addition proofed to remain at stable levels throughout the disease course. Implementation of CSF NfL as a potential read-out for future therapeutic trials in ALS is currently constrained by its demonstrated susceptibility to (pre-)analytical variations. Here we show that the D50 model enables the discovery of correlations between clinical characteristics and CSF analytes and can be recommended for future studies evaluating potential biomarkers.

## Introduction

Amyotrophic lateral sclerosis (ALS) is a fatal neurodegenerative disorder that is predominately characterized by the progressive loss of motor neuron function. The clinical presentation of the disease varies significantly among patients, with atrophy and weakness as well as spasticity and fasciculations in limb, bulbar, and thoracic muscles. Despite constant efforts to develop new disease-modifying therapies, survival for most patients with ALS is still restricted to 2–5 years after symptom onset (Paganoni et al., 2014).

As phenotypic variability and disease course variability represent major constraints to clinical management and therapeutic trials in ALS, the search for biomarkers that can accurately predict progression is a current research priority. Previous therapeutic trials predominantly employed clinical measures such as long-term survival rates and linearly approximated declines of the ALS Functional Rating Scale-Revised (ALSFRS-R) as outcome measures (Petrov et al., 2017). The detection of significant treatment effects in these trials requires large sample sizes and consumes time and resources, which could be improved by specific pharmacodynamic or prognostic/predictive biomarkers. The importance of such biomarkers has been underlined in the recently revised Airlie House consensus criteria for clinical trial development in ALS (Van Den Berg et al., 2019).

Cerebrospinal fluid (CSF) neurofilaments are promising candidate biomarkers with prognostic implications in ALS. Neurofilaments constitute the main structural components of motor axons. Following neuroaxonal damage, increased concentrations of neurofilament light chain (NfL) and phosphorylated neurofilament heavy chain (pNfH) have been reported in both CSF and blood in various neurologic disorders (Khalil et al., 2018). While CSF pNfH has demonstrated greater diagnostic accuracy (Poesen et al., 2017), the concentration of NfL in the CSF of ALS patients reportedly correlates with both survival (Zetterberg et al., 2007; Pijnenburg et al., 2015; Gaiani et al., 2017; Gong et al., 2018; Illán-Gala et al., 2018; Rossi et al., 2018; Scarafino et al., 2018; Schreiber et al., 2018; Kasai et al., 2019; Abu-Rumeileh et al., 2020) and the disease progression rate (Tortelli et al., 2012; Lu et al., 2015; Menke et al., 2015; Steinacker et al., 2016, 2018b; Gaiani et al., 2017; Poesen et al., 2017; Andres-Benito et al., 2018; Gong et al., 2018; Rossi et al., 2018; Scarafino et al., 2018; Schreiber et al., 2018; Abu-Rumeileh et al., 2020). These findings suggest that CSF NfL concentrations at baseline may allow early stratification of patients in clinical trials according to anticipated progressiveness, thereby reducing clinical heterogeneity and enabling the detection of significant treatment effects even in smaller ALS patient cohorts.

However, the exact role of NfL in ALS is not yet entirely understood, and several challenges hamper its routine use as a biomarker in clinical trials. CSF NfL has been reported to correlate not only with the rate of disease progression but also with the clinical status at the time of lumbar puncture, as assessed by clinical scores or imaging measures of disease severity (Tortelli et al., 2012; Steinacker et al., 2016, 2018b; Gong et al., 2018; Scarafino et al., 2018). This raises the question of whether CSF NfL reflects cumulative neuroaxonal damage rather than the rate of neuroaxonal breakdown. As patients with faster disease courses have typically reached a more advanced disease stage at the time of sampling (sampling shift), these factors are inextricably intertwined in ALS patient cohorts. The temporal profile of CSF NfL throughout the disease course remains to be more precisely elucidated. The few available longitudinal studies on CSF NfL in patients with ALS comprised rather small sample sizes and reported inconsistent results (Lu et al., 2015; Steinacker et al., 2016; Poesen et al., 2017; Skillbäck et al., 2017; Benatar et al., 2018; Huang et al., 2020). Furthermore, the concentration of NfL in the CSF may be influenced by several other factors, including the presence of frontotemporal dementia (FTD) (Illán-Gala et al., 2018; Steinacker et al., 2018a), different ALS genotypes (Zetterberg et al., 2007; Huang et al., 2020), or the predominant affection of upper motor neurons (UMNs) rather than lower motor neurons (LMNs) (Rosengren et al., 1996; Gaiani et al., 2017; Schreiber et al., 2018).

An additional concern is the interlaboratory variation of CSF NfL measurements (Oeckl et al., 2016; Gray et al., 2020), as validation of biomarkers and translation into clinical trials require multicenter confirmation.

In an attempt to address the mentioned uncertainties regarding the prognostic role of CSF NfL in ALS, we applied the D50 disease progression model (Poesen et al., 2017; Prell et al., 2019; Steinbach et al., 2020) in a large-scale cross-sectional cohort. As the model addresses the phenotypic heterogeneity inherent to the disease and reduces the noise associated with the ALSFRS-R, this approach may help uncover the effect of disease aggressiveness on CSF neurofilament levels in a clinically diverse ALS patient cohort, while simultaneously controlling for the potential influence of disease accumulation at the time of sampling.

## Materials and Methods

### Participants

All participants were recruited from the neuromuscular center at the University Hospital of Jena, Germany, between 2013 and 2020. The participants provided written and informed consent prior to study commencement, and the study was approved by the local ethics committee (Nr 3633-11/12). Two hundred seventy-three participants with available CSF NfL measurements were identified from our local specialized neuromuscular disease database. Based on clinical disease histories, a total of 238 participants could be allocated to one of the four following condition groups: (a) non-neurological controls (*n* = 15); (b) disease controls (*n* = 56), suffering from neurologic disorders other than ALS; (c) ALS mimics (*n* = 11), with other conditions that shared symptomatology with an ALS disease course; and (d) patients with ALS (*n* = 156) (Supplementary Table 1). Of the initial 185 ALS patients, 29 patients were excluded, either because fewer than two ALSFRS-R assessments were available (*n* = 16), or because the Gold Coast criteria for the diagnosis of ALS (Shefner et al., 2020) were not fulfilled (*n* = 13). From a total of 62 disease controls, six were excluded because of an uncertain diagnosis (*n* = 5) or acute intracranial bleedings (*n* = 1).

### Diagnosis and Phenotypic Characterization of Patients With ALS

One hundred fifty-six patients fulfilled the recently defined Gold Coast criteria for the diagnosis of ALS at the time of CSF sampling (Shefner et al., 2020) and had a minimum of two ALSFRS-R scores obtained throughout the disease course. According to the revised El Escorial criteria at the time of lumbar puncture, ALS patients had either suspected, possible, laboratory-supported probable, probable, or definite ALS (Brooks et al., 2000). According to the evaluation of the entire disease history of these patients, they presented with one of the following clinical phenotypes: classic, bulbar, pyramidal, flail arm, flail leg, or respiratory or pure LMN, according to the classification by Chió et al. in 2011 (Chiò et al., 2011). The diagnosis of clinically overt frontotemporal dementia (FTD) was made by experienced neurologists at the University Hospital Jena based on clinical observations. All 6 patients diagnosed with FTD fulfilled the original Strong diagnostic criteria for the diagnosis of FTD (Strong et al., 2009, 2017).

We also estimated the number of regions (bulbar, cervical, thoracic, or lumbar) with UMN and/or LMN involvement at the time of CSF sampling. The four regions were evaluated clinically and electromyographically according to the revised El Escorial and Awaji criteria (Brooks et al., 2000; de Carvalho et al., 2008). Hence, ALS patients were divided into categories of one (none or one region), two (two regions), or three (three or four regions) regions affected by UMN and/or LMN degeneration. ALS patients were also classified according to (a) the King’s staging system (Roche et al., 2012) and (b) the Milano–Torino Staging System (MiToS) (Chio et al., 2015), both calculated using the ALSFRS-R closest to the time of CSF sampling. The King’s staging system allocates patients to stages I (involvement of one clinical region) to IV (respiratory or nutritional failure), whereas the MiToS System describes stages 0 (functional involvement) to IV (loss of independence in four domains) (Roche et al., 2012; Chio et al., 2015).

### The D50 Disease Progression Model

**FIGURE 1:**
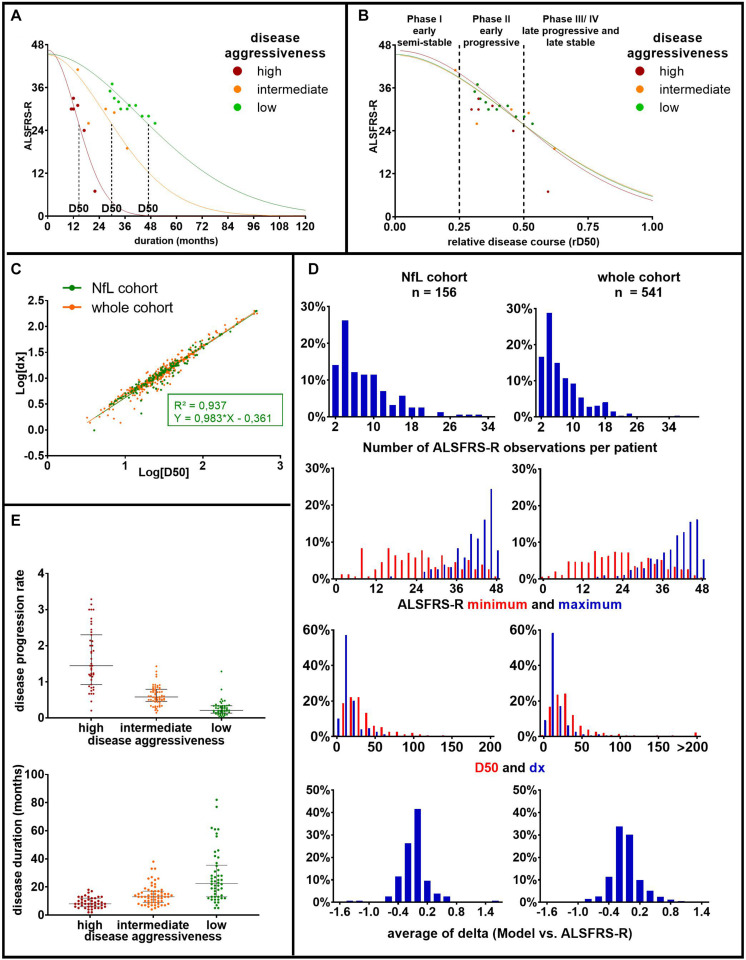
Principles and parameters of the D50 model: **(A)** based on consecutively obtained ALSFRS-R scores (dots), a sigmoidal functional decline curve is calculated. The value D50 depicts the individual time in months since symptom onset until halved functionality, indicating the overall disease aggressiveness of each individual patient. The curves represent three example patients with either high (D50 = 14.56 months, in red), intermediate (D50 = 29.88 months, in orange), or low disease aggressiveness (D50 = 46.84 months, in green). **(B)** Normalization of patient’s individual sigmoidal curves by D50 yields the parameter relative D50 (rD50). rD50 describes the individual disease covered and facilitates the comparison of vastly differing progression types. **(C)** The parameter D50 linearly correlates with the time constant of ALSFRS-R decline (dx) in this, as well as in other ALS cohorts. Thus, D50 alone can be used to describe patients’ disease aggressiveness. **(D)** Histograms of pertinent disease variables for the patients of the current cohort (NfL cohort), as well as all ALS patient data available in our center (whole cohort). It illustrates that the current cohort well coincides with the entire ALS patient cohort treated at our center. **(E)** Scatterplots of patients’ disease progression rate and disease duration at the time of sampling, subdivided by the three D50 subgroups in our cohort: (a) high (0 ≤ D50 < 20, in red), (b) intermediate (20 ≤ D50 < 40, in orange), and (c) low (40 ≤ D50, in green) disease aggressiveness. It illustrates large variations of the disease progression rate, especially within the high aggressive subgroup. Bars indicate median and interquartile range. ALS, amyotrophic lateral sclerosis; ALSFRS-R, Amyotrophic Lateral Sclerosis Functional Rating Scale-Revised; rD50, relative D50; NfL, neurofilament light chain.

To assess the impact of clinical characteristics of patient’s ALS disease course on CSF NfL, the D50 disease progression model was applied (Poesen et al., 2017; Prell et al., 2020; Steinbach et al., 2020). The D50 model was chosen because it provides quantitative measures of disease aggressiveness, distinct from parameters of disease accumulation, and thus provides a framework to interpret associations with any biomarker (Figure 1). The model uses regularly assessed ALSFRS-R scores of each individual patient to calculate a sigmoidal state transition from full health to functional loss. Here, we applied an adaptation of the model that allows a variable presymptomatic phase of supratotal functionality up to 6 months prior to symptom onset. This approach accounts for the known uncertainties in the exact time point of first symptoms as reported by the patients, as well as a presymptomatic breakdown of motoric functional reserves. The resulting sigmoidal curve can be characterized by (a) the value D50, which describes the time taken in months from symptom onset to reach halved functionality, and (b) the dx, the time constant of functional decline. Because dx and D50 correlate linearly (Figure 1C), the D50 value alone provides a meaningful descriptor of patients’ overall disease aggressiveness.

The ALS patient cohort could thus be divided into three groups of (a) high (0 ≤ D50 < 20), (b) intermediate (20 ≤ D50 < 40), and (c) low (40 ≤ D50) disease aggressiveness. A normalization of patient’s real-time sigmoidal disease trajectory to D50 yields the parameter relative D50 (rD50), an open-ended reference scale where 0 signifies disease onset and 0.5 the time point of halved functionality (Figure 1B). The rD50 provides an individualized time scale of accumulated disease (independent of disease aggressiveness) and was calculated for the individual time point of lumbar puncture. Patients with ALS could thus also be grouped into one of the following three phases: the early semistable phase I (0 ≤ rD50 < 0.25), the early progressive phase II (0.25 ≤ rD50 < 0.5), and the late progressive and stable phase (III/IV) (0.5 ≤ rD50).

For comparability with former studies, we also calculated the more traditionally used linear disease progression rate at the time of CSF sampling, defined as (48 - ALSFRS-R at sampling)/disease duration in months (Figure 1E).

### CSF Collection and Analysis

All CSF samples were collected via lumbar puncture at the Department of Neurology, Jena University Hospital. The samples were centrifuged, aliquoted, and stored at −80°C within 2 h after lumbar puncture. NfL concentration was assessed using the commercially validated IBL International enzyme-linked immunosorbent assay (ELISA) kit at two different European laboratories: (a) in Germany (*n* = 140) and (b) in Belgium (*n* = 99). All samples and standards were assayed in duplicate and in accordance with manufacturer instructions; intra-assay and interassay variations were ≤10%, and ≤20%, respectively.

### Statistical Analysis

Statistical analyses were performed using the SPSS^®^ Statistics software program (v27.0.0.0 IBM^®^, Chicago, IL, United States). For graphical representation of data, GraphPad Prism was used (v9.0.0 for Windows, GraphPad Software, San Diego, CA, United States). Normal distribution of variables was assessed with the Shapiro–Wilk test. Normal distribution of NfL concentration was achieved via log transformation, and log[NfL] was used for parametric testing. Two-sample *t*-tests were used for comparison of Log[NfL] concentrations between ALS patients and control groups. Receiver operating characteristic curves were used to calculate the sensitivity and specificity of CSF NfL for differentiating ALS from the control groups. The optimal cutoff was calculated with the Youden Index.

To evaluate differences in NfL concentrations between different ALS subgroups, a one-way analysis of covariance (ANCOVA) was applied, followed by pairwise *post hoc* tests with Bonferroni correction. For the comparison of low, intermediate, and high disease aggressiveness subgroups, the following covariates were applied: age, sex, FTD, laboratory of NfL measurement, clinical phenotype, and disease phase.

In our ALS cohort, a significant sampling shift occurred, which was previously observed in other cohorts analyzed using the D50 model (Table 1): patients with slow and intermediate progression were still in the earlier phases of the disease at the time of sampling, whereas patients with fast progression had already reached later disease phases by the time they were referred to our center, and lumbar puncture was performed. Therefore, the covariate disease phase did not meet the assumption for ANCOVA of homogenous distribution over the three subgroups. We therefore conducted an additional ANCOVA in a filtered ALS cohort, in which patients of all disease phases were equally distributed throughout the three aggressiveness subgroups (Supplementary Table 2).

**TABLE 1:** Demographic and clinical data for patients with ALS (*n* = 156).

		Disease aggressiveness			*p*
		
		High (D50 < 20)	Intermediate (20 ≤ D50 < 40)	Low (D50 ≥ 40)	
*n*		43	61	52	

**Neurofilament light chain (NfL) measurements**					

NfL (pg/mL)^$^		14,500.0 (7,883.0–24,680.0)	8,959.67 (4,410.5–12,157.5)	4,426.69 (2,879.5–7,333.5)	<0.001*
Laboratory: Germany/Belgium		29/14 67.4%/32.6%	35/26 57.4%/42.6%	40/12 76.9%/23.1%	0.253

**Demographics**					

Age at lumbar puncture		64.3 ± 9.54	63.33 ± 10.47	61.42 ± 10.95	0.384
Gender: Male/female		23/20 53.5%/46.5%	37/24 60.7%/39.3%	34/18 65.4%/34.6%	0.514

**D50 disease progression model parameters**					

D50^$^		13.62 (9.40–16.14)	28.81 (23.07–31.73)	62.58 (46.12–96.61)	−
rD50^$^		0.37 (0.23–0.45)	0.23 (0.17–0.32)	0.18 (0.10–0.32)	<0.001*
Phase	I (rD50 < 0.25)	11 (25.6%)	32 (52.2%)	33 (63.5%)	0.001*
	II (0.25 ≤ rD50 < 0.5)	27 (62.8%)	27 (44.3%)	19 (36.5%)	
	III/IV (rD50 ≥ 0.5)	5 (11.6%)	2 (3.3%)	0 (0%)	

**Traditional disease metrics**					

ALSFRS-R at lumbar puncture^$^		35 (29–40)	41 (38.50–44)	42 (39.25–45.75)	<0.001*
Disease progression rate^$^		1.64 (1.05–2.30)	0.60 (0.46–0.74)	0.21 (0.13–0.33)	<0.001*
Disease duration at lumbar puncture (mo)^$^		8 (2–18)	13 (4–38)	23.50 (5–212)	<0.001*
King’s stage	I	10 (23.3%)	20 (32.8%)	21 (40.4%)	0.008*
	II	11 (25.6%)	24 (39.9%)	24 (46.2%)	
	III	17 (39.5%)	12 (19.7%)	7 (13.5%)	
	IV a	3 (7%)	1 (1.6%)	0 (0%)	
	IV b	2 (4.7%)	4 (6.6%)	0 (0%)	
	V	0 (0%)	0 (0%)	0 (0%)	
MiToS stage	0	21 (48.8%)	52 (85.2%)	46 (88.5%)	<0.001*
	I	18 (41.9%)	7 (7%)	6 (11.5%)	
	II	4 (9.3%)	2 (3.3%)	0 (0%)	
	III–V	0 (0%)	0 (0%)	0 (0%)	
ALS phenotype	Classic	21 (48.8%)	38 (62.3%)	33 (63.5%)	0.058
	Bulbar	18 (41.9%)	19 (31.1%)	9 (17.3%)	
	Pyramidal	3 (7%)	4 (6.6%)	5 (9.6%)	
	Respiratory	1 (2.3%)	0 (0%)	0 (0%)	
	Flail arm	0 (0%)	0 (0%)	3 (5.8%)	
	Flail leg	0 (0%)	0 (0%)	1 (1.9%)	
	Pure LMN	0 (0%)	0 (0%)	1 (1.9%)	
Revised El Escorial criteria	Definitive	10 (23.3%)	3 (4.9%)	1 (1.9%)	<0.001*
	Probable	22 (51.2%)	33 (54.1%)	19 (36.5%)	
	Laboratory-supported probable	8 (18.6%)	20 (32.8%)	18 (34.6%)	
	Possible	3 (7%)	3 (4.9%)	9 (17.3%)	
	Suspected	0 (0%)	2 (3.3%)	5 (9.6%)	
Presence of FTD: yes/no		2/41 4.7%/95.3%	3/58 4.9%/95.1%	1/51 1.9%/98.1%	0.671
Riluzole treatment: yes/no	42/1 97.7%/2.1%	60/1 98.4%/1.6%	49/3 94.2%/5.8%	0.671

Continuous variables with normal distribution are expressed as mean with standard deviation. Categorical variables are expressed as number and percentage. For the comparison of demographic and clinical variables among the three aggressiveness subgroups, analyses of variance, Kruskal–Wallis tests, χ^2^ tests, or Fisher–Freeman–Halton exact tests were applied where appropriate.^$^Non-parametric nominal variables, represented as median and interquartile range.*Statistical significance at *p* < 0.05.ALS, amyotrophic lateral sclerosis; ALSFRS-R, Revised ALS Functional Rating Scale; FTD, frontotemporal dementia; LMN, lower motor neuron; MiToS, Milano–Torino Staging System; NfL, neurofilament light chain; rD50, relative D50.

A one-way ANCOVA was conducted to compare CSF Log[NfL] concentration of the three disease phases, while controlling for disease aggressiveness, FTD, clinical phenotype, age, gender, and laboratory of measurement.

Linear regression analysis and Spearman correlation was used to assess correlations between NfL, D50, and rD50 at the time of sampling. Pearson correlation was used to assess correlation between paired Log[NfL] measurements from the two centers in Germany and Belgium. Differences between CSF NfL concentrations of ALS patients with and without FTD were tested with a Mann–Whitney *U* test.

For survival analyses, ALS patients were divided into three groups with low (Log[NfL] < 3.651), intermediate (3.651 ≤ Log[NfL] < 4.149), and high (4.149 ≤ Log[NfL]) CSF NfL concentrations, with cutoffs derived from the estimated marginal means of our previously described ANCOVA (comparing disease aggressiveness subgroups). The Kaplan–Meier method was used for survival analyses, and subgroups were compared with a log–rank test. 97 patients (13 with low, 51 with intermediate, and 33 with high CSF NfL levels) reached the endpoint death or tracheostomy, whereas the remaining 59 patients were censored. Statistical significance was defined as *p* < 0.05.

## Results

### Diagnostic Performance of CSF NfL in ALS

**FIGURE 2:**
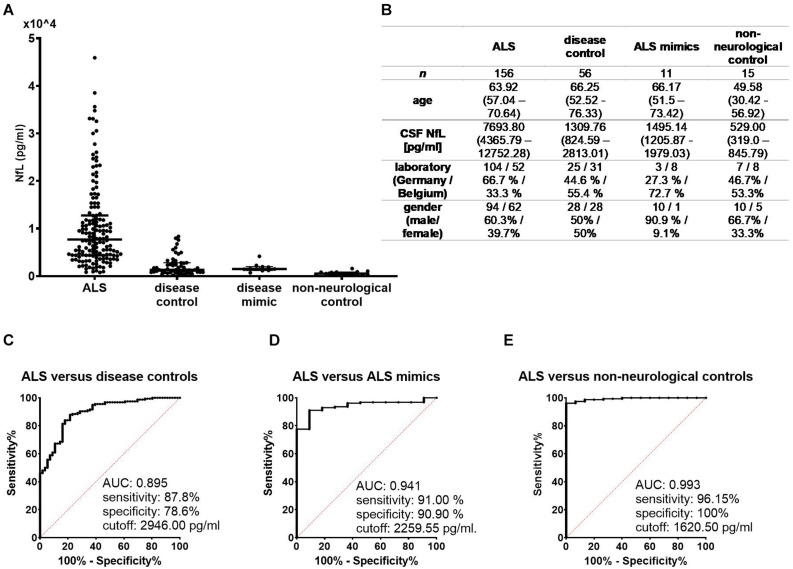
**(A)** NfL concentrations in cerebrospinal fluid were significantly higher in the ALS group compared to each control group (*p* < 0.001 for each pairwise comparison). **(B)** Demographic and clinical data of the four condition groups are expressed as either median with interquartile range or as number and percentages. Receiver operating characteristic curves illustrate the diagnostic performance of NfL in distinguishing ALS from disease controls **(C)**, ALS mimics **(D)**, and non-neurological controls **(E)**. ALS, amyotrophic lateral sclerosis; AUC, area under the curve; NfL, neurofilament light chain.

Cerebrospinal fluid Log[NfL] levels were significantly higher in the ALS group (mean = 3.87, SD = 0.37) as compared to the non-neurological control (mean = 2.72, SD = 0.27, *p* < 0.001), disease control (mean = 3.18, SD = 0.38, *p* < 0.001), and ALS mimic groups (mean = 3.20, SD = 0.19, *p* < 0.001). When distinguishing ALS from disease controls, the area under the curve (AUC) was 0.895 (0.849–0.9413), sensitivity was 87.8%, and specificity was 78.6% at a cutoff of 2,946.00 pg/mL. For the differentiation between ALS and ALS mimics, the AUC was 0.941 (0.897–0.985), sensitivity was 91.0%, and specificity was 90.9% at a cutoff of 2,259.55 pg/mL. A cutoff of 1,620.5 pg/mL distinguished ALS patients from non-neurological controls with a sensitivity of 96.15% and specificity of 100% [AUC = 0.993 (0.984–1.002)] (Figure 2).

Cerebrospinal fluid NfL levels did not significantly differ between different ALS phenotypes [*F*(6,149) = 0.925, *p* = 0.479]. Patients with FTD had significantly higher CSF NfL levels relative to those without FTD (*U* = 208.0, *Z* = -2.23, *p* < 0.05).

### Cohort of Patients With ALS

Detailed demographic and clinical data of ALS patients are shown in Table 1. Age, gender, and laboratory of analysis did not significantly differ between patients with high, intermediate, or low disease aggressiveness. The rD50 at the time of lumbar puncture, as well as the rD50-derived disease phase, showed significant differences between these three subgroups, as patients with lower disease aggressiveness were still in the earlier phases of the disease due to the sampling shift. Accordingly, the more traditionally used disease metrics, namely, the ALSFRS-R, the King’s and MiToS stages, the stage of diagnostic certainty according to the revised El Escorial criteria (Brooks et al., 2000), the disease duration (time between symptom onset and lumbar puncture), and the disease progression rate, differed significantly between the three subgroups. Other disease characteristics, such as ALS phenotype, presence of FTD, or Riluzole intake, were homogenously distributed throughout the three subgroups.

### CSF NfL Predicts Disease Aggressiveness

**FIGURE 3:**
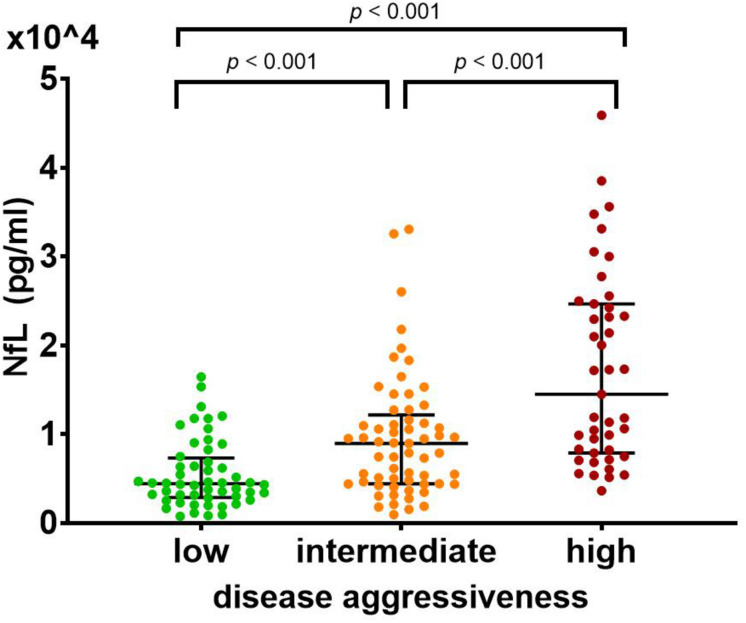
CSF NfL differs significantly between patients with (a) high (0 ≤ D50 < 20, in red), (b) intermediate (20 ≤ D50 < 40, in orange), and (c) low (40 ≤ D50, in green) disease aggressiveness. This effect remained significant after controlling for clinical phenotype, presence of frontotemporal dementia, age, gender, disease phase, and laboratory of measurement in an ANCOVA (*p* < 0.001). *Post hoc* pairwise comparisons of the estimated marginal means confirmed an increase of NfL levels with increasing disease aggressiveness (low: 4,477.13, intermediate: 7,194.49, high: 14,092.89; *p* < 0.001 for all pairwise comparisons). Bars indicate median and interquartile range. ANCOVA, analysis of covariance; NfL, neurofilament light chain.

The ANCOVA showed a significant main effect for CSF Log[NfL] (pg/mL) concentrations of the three disease aggressiveness subgroups [*F*(2,147) = 30.055, *p* < 0.001]. *Post hoc* pairwise comparisons of the estimated marginal means showed that CSF Log[NfL] was highest in the highly aggressive disease subgroup (mean = 4.149), lower in the intermediate aggressiveness subgroup (mean = 3.857) and lowest in patients with low disease aggressiveness (mean = 3.651; *p* < 0.001 for all pairwise comparisons) (Figure 3). The covariates age, [*F*(1,147) = 12.451, *p* < 0.001], laboratory of analysis [*F*(1,147) = 13.748, *p* < 0.001], and FTD [*F*(1,147) = 6.176, *p* = 0.014] were also significantly related to CSF Log[NfL], whereas gender, disease phenotype, and phase showed no impact.

The main effect of disease aggressiveness on Log[NfL] remained in a similar ANCOVA for the filtered cohort (with homogenous distribution of disease phases over the three aggressiveness subgroups). Most importantly, the disease phase did not have a significant effect on Log[NfL] concentrations (Supplementary Table 3).

**FIGURE 4:**
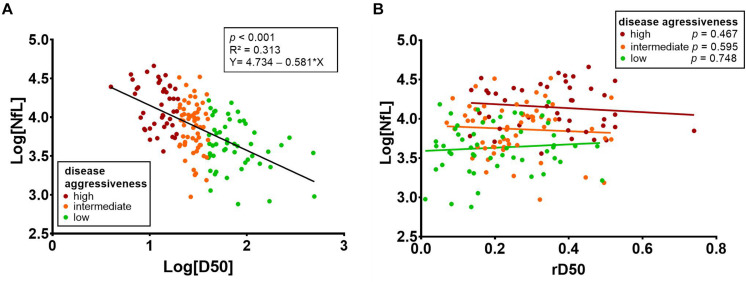
**(A)** There was a negative correlation between the D50 parameter and CSF NfL (*p* < 0.001, ρ = -0.553). Linear regression analysis showed that 31.3% of the variation in CSF NfL can be explained by the D50 parameter (*R*^2^ = 0.313, Log[NfL] = 4.734 - 0.581 × Log[D50]; *p* < 0.001). **(B)** Stratification of patients into the three aggressiveness subgroups (based on D50) reveals that there is no significant correlation of CSF NfL with rD50. CSF, cerebrospinal fluid; rD50, relative D50; NfL, neurofilament light chain.

There was a negative correlation between the D50 parameter and CSF NfL (*p* < 0.001, ρ = -0.553) (Figure 4A). The linear regression analysis showed that 31.3% of the variation in CSF NfL can be explained by the D50 parameter (*R*^2^ = 0.313, Log[NfL] = 4.734 - 0.581 × Log[D50], *p* < 0.001). This correlation remained significant when analyzing patients in disease phases I and II separately (phase I: n = 76, *p* < 0.001, ρ = -0.528, phase II: *n* = 73, *p* < 0.001, ρ = −0.521). Patients in phase III/IV showed a similar tendency of negative correlation, but did not reach statistical significance, most likely due to the small sample size (*n* = 7, *p* < 0.337, ρ = −0.429).

### CSF NfL Is Independent of Disease Phase and Number of Affected Regions

There was no significant main effect of disease phase on Log[NfL] concentrations [*F*(2,147) = 1.692, *p* = 0.188] in the respective ANCOVA, but the covariates disease aggressiveness *F*(1,147) = 61.032, *p* < 0.001), age [*F*(1,147) = 13.603, *p* < 0.001], laboratory of analysis [*F*(1,147) = 13.927, *p* < 0.001], and FTD [*F*(1,147) = 6.284, *p* = 0.013] showed a significant impact.

For the whole ALS patient cohort, a correlation between CSF NfL and rD50 was noted (*p* = 0.005, ρ = 0.224); however, this did not retain significance when stratifying patients into the three D50 subgroups (Figure 4B). This calculated correlation of CSF NfL with rD50 is thus likely attributable to the aforementioned cohort-specific intercorrelation between the parameters rD50 and D50, resulting from the sampling shift (*p* < 0.001, ρ = -0.432) (Supplementary Figure 1).

There were no significant differences in the CSF Log[NfL] concentration when stratifying patients according to the number of regions with UMN [*F*(2,153) = 2.858, *p* = 0.060] or LMN [*F*(2,153) = 0.659, *p* = 0.519] involvement at the time of sampling. Also, in combination, the number of regions with UMN and/or LMN affection did not have a significant effect on the CSF Log[NfL] concentrations [*F*(2,153) = 1.403, *p* = 0.249] (Supplementary Table 4).

### CSF NfL Predicts Survival in Patients With ALS

**FIGURE 5:**
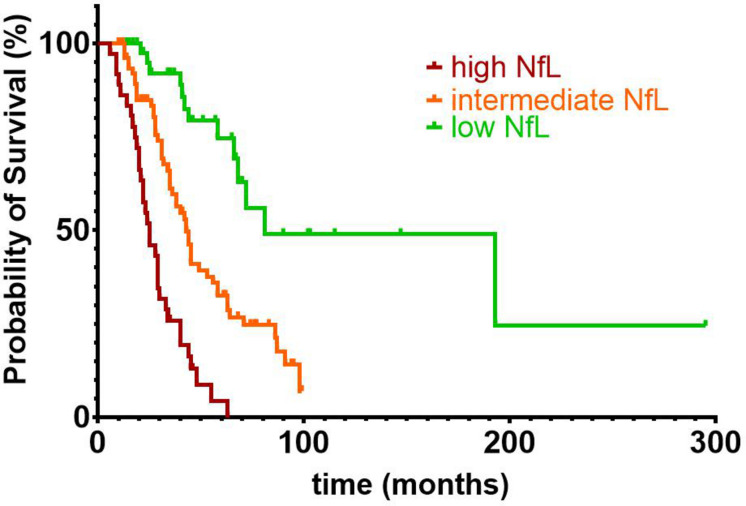
Kaplan–Meier survival curves and log-rank test showed significant differences in survival [χ^2^(2) = 56.505, *p* < 0.001], when trichotomizing the ALS patients into groups with high (*n* = 36), intermediate (*n* = 77), and low (*n* = 43) CSF NfL concentrations. Estimated marginal means of the previously described analysis of covariance were used for the subdivision of ALS patients into groups with high (Log[NfL] > 4.149), intermediate (3.651 < Log[NfL] < 4.149), and low (Log[NfL] < 3.651) CSF NfL concentrations. Of the 156 ALS patients included in the survival analysis, 97 patients (13 with low, 51 with intermediate, and 33 with high CSF NfL levels) reached the endpoint death or tracheostomy, whereas the remaining 59 patients were censored. ALS, amyotrophic lateral sclerosis; CSF, cerebrospinal fluid; NfL, neurofilament light chain.

Kaplan–Meier survival curves and log–rank tests showed significant differences in survival [χ^2^(2) = 56.505, *p* < 0,001], when trichotomizing the ALS patients into groups with high (*n* = 36), intermediate (*n* = 77), and low (*n* = 43) CSF Log[NfL] concentrations based on disease aggressiveness–adjusted marginal means (Figure 5).

### Interlaboratory Variation and Paired Sample Comparison

Cerebrospinal fluid samples from 57 patients with ALS were pairwise analyzed in both laboratories. The mean coefficient of variation of CSF NfL measurements between laboratories was 21.19% (SD = 24.75) for these 57 samples. There was a strong positive correlation between paired CSF Log[NfL] measurements from both laboratories (*r* = 0.918, *p* < 0.001) (Supplementary Figure 2).

## Discussion

In the present study we showed that CSF NfL levels in ALS patients significantly differ between patients according to their D50-derived disease aggressiveness. In addition to interlaboratory variation, significant effects for age and FTD on CSF NfL concentrations were also noted. However, the rD50 value and the derived disease phase did not influence NfL levels.

Associations between CSF NfL and the disease progression rate in ALS have been previously proposed (Tortelli et al., 2012; Lu et al., 2015; Menke et al., 2015; Steinacker et al., 2016, 2018b; Poesen et al., 2017; Andres-Benito et al., 2018; Gong et al., 2018; Scarafino et al., 2018; Schreiber et al., 2018; Abu-Rumeileh et al., 2020). However, the interpretation of these analyses remained constrained, because of the incomplete evaluation of confounding factors that influence NfL levels and/or the lack of longitudinal validation studies. Moreover, former results were limited to correlations with single disease metrics, such as the disease progression rate or the ALSFRS-R. This neglects the huge interindividual heterogeneity of disease courses in ALS, requiring a quantifiable framework within which to interpret patients’ individualized disease trajectories and putative biomarkers.

We therefore applied the D50 model that provides quantifications for both measures of disease aggressiveness (D50), as well as the amount of disease covered (rD50, phase) at the time of CSF sampling to generate a large-scale pseudo–longitudinal analysis. This allowed us to demonstrate that CSF NfL is increased in patients with higher disease aggressiveness, even after adjustment for interlaboratory variation, age, gender, ALS phenotype, presence of FTD, and disease phase at the time of sampling. Former studies showed correlations between CSF NfL and linearly approximated progression rates. Most of these studies calculated the decline in ALSFRS-R from symptom onset until CSF sampling (Tortelli et al., 2012; Lu et al., 2015; Menke et al., 2015; Steinacker et al., 2016, 2018b; Poesen et al., 2017; Andres-Benito et al., 2018; Gong et al., 2018; Scarafino et al., 2018; Schreiber et al., 2018) or from symptom onset until disease diagnosis (Gaiani et al., 2017; Rossi et al., 2018). However, linear mixed-effects models using consecutively obtained ALSFRS-R scores have also been used to demonstrate associations with CSF NfL levels (Huang et al., 2020).

All these studies presume a linear decline of the ALSFRS-R score over time, despite prior observations that the rate of decline varies with disease progression and follows a curvilinear course (Gordon et al., 2010). Moreover, the calculation of progression rates based on a single score is highly susceptible to the known intrarater and interrater variability associated with ALSFRS-R assessments (Bakker et al., 2020). We therefore propose that the D50 model provides a more accurate representation of clinical progression, as it calculates an individualized sigmoidal curve of functional deterioration for each patient (Poesen et al., 2017; Prell et al., 2020; Steinbach et al., 2020).

The association between CSF NfL and survival in our ALS cohort further substantiates the ability of this biomarker to reflect prognosis in these patients and is in accordance with previous studies on CSF NfL and survival in ALS (Zetterberg et al., 2007; Pijnenburg et al., 2015; Gaiani et al., 2017; Gong et al., 2018; Illán-Gala et al., 2018; Rossi et al., 2018; Scarafino et al., 2018; Schreiber et al., 2018; Kasai et al., 2019).

The lack of a significant effect of the disease phase on NfL levels indicates that CSF concentrations remain longitudinally stable throughout the disease course. This suggests that any baseline NfL measurement is able to predict patients’ disease aggressiveness, independent of the time point of CSF sampling. While longitudinal studies on CSF NfL concentrations in ALS would be best suited to support this observation, these are scarce and mostly comprise small numbers of patients. Some longitudinal studies reported rather stable levels throughout the disease course (Benatar et al., 2018; Huang et al., 2020), but slightly decreasing (Steinacker et al., 2016) and increasing concentrations in specific subpopulations of ALS patients (Lu et al., 2015; Poesen et al., 2017; Skillbäck et al., 2017) have been reported as well.

Several longitudinal studies following presymptomatic ALS-causing mutation carriers until the occurrence of manifest disease have aided in the understanding of the temporal profile of CSF NfL concentrations (Benatar et al., 2018, 2019). In these studies, while asymptomatic patients initially had CSF NfL concentrations similar to controls, increases were observed more than a year prior to phenoconversion (defining a presymptomatic stage) (Benatar et al., 2018, 2019). Recent findings also suggest that the duration of this presymptomatic stage may differ in accordance to the patient’s survival (Benatar et al., 2019).

Studies have also reported correlations between CSF NfL and the ALSFRS-R at the time of sampling (Tortelli et al., 2012; Steinacker et al., 2016, 2018b; Gong et al., 2018; Scarafino et al., 2018), suggesting that NfL reflects cumulative neuroaxonal damage (disease accumulation) rather than the rate of neuronal breakdown (i.e., aggressiveness). We would like to emphasize that both aspects (disease accumulation and aggressiveness) are inherently interdependent in ALS cohorts, as patients with higher disease aggressiveness typically have reached a more advanced disease phase at the time of referral to ALS centers (sampling shift).

Moreover, most studies on neurofilaments performed univariate analyses to assess associations between clinical metrics and CSF NfL concentrations and neglected possible confounders. In one multivariate study by Gaiani et al., a repeated-measures ANCOVA was performed to investigate the effects of CSF NfL, ALS subtype, age, disease progression rate, gender, and cognitive impairment on longitudinal ALSFRS-R and MiToS scores. It was shown that all covariates, except cognitive impairment, exhibited significant effects on the functional-impairment scores (Gaiani et al., 2017). Another recent study investigated the effect of several clinical predictors of prognosis (including age, sex, C9ORF72 status, site of onset, baseline ALSFRS-R, and disease progression rate) on the ALSFRS-R slope in a multivariate model and demonstrated that serum NfL adds prognostic value to the model, but a comparable analysis on CSF NfL was lacking (Benatar et al., 2020). However, to the best of our knowledge, no former study has used multivariate analysis to probe the impact of several disease-specific variables on CSF NfL levels in ALS.

The present study provides strong evidence that CSF NfL reflects overall disease aggressiveness in ALS, independent of disease accumulation. This supports the concept that NfL and, more broadly, neurofilament proteins reflect disease activity. They might be directly linked to the pathophysiological process itself rather than being a collateral by-product of neuronal degeneration (Julien, 2001; Petzold, 2005). NfL may thus be used to directly monitor the therapeutic effects of neuroprotective or other disease-modifying drugs in clinical trials, where a positive therapeutic effect may be reflected by a reduction in the rate of release of NfL into the CSF. There is currently a growing momentum for the implementation of neurofilaments as secondary endpoints in such trials, with first promising findings in ALS (Miller et al., 2020), as well as spinal muscular atrophy patients (Olsson et al., 2019) under disease-modifying treatments. Our data suggest that CSF NfL represents a suitable monitoring biomarker for ALS that might be sensitive to therapeutic regimens aimed at decreasing disease aggressiveness. However, future longitudinal studies would be needed to assess its potential as an outcome measure for long-term treatment in ALS.

Besides disease aggressiveness, three covariates exhibited statistically significant effects on CSF NfL levels of ALS patients. In accordance with previous studies, age showed a positive association with CSF NfL (Vågberg et al., 2015; Gong et al., 2018; Steinacker et al., 2018b; Sun et al., 2020). This most likely reflects the degenerative process in the brain associated with normal aging, which leads to a slowly progressive rise of neurofilaments in the CSF. The ELISAs were performed in two different laboratories, and the site of analysis showed a statistically significant impact on NfL concentrations in the CSF. Stability issues of NfL measurements have been reported in previous multicentric studies on NfL and have been related, *inter alia*, to differences in perianalytical procedures (Petzold et al., 2010; Oeckl et al., 2016; Gray et al., 2020). This underlines the necessity for the implementation of standard operating procedures and round-robin tests. However, the coefficient of variation between measurements of both participating laboratories in this study was lower than previously reported for the same ELISA kit (Petzold et al., 2010; Miller et al., 2016; Gray et al., 2020), and the interlaboratory variations did not obscure the highly significant effect of disease aggressiveness on CSF NfL. Higher NfL levels in ALS patients with a concomitant diagnosis of FTD in our study are also in accordance with previous reports (Illán-Gala et al., 2018; Steinacker et al., 2018a).

We did not find a significant association between CSF NfL and the number of regions affected by UMN or LMN degeneration at the time of CSF collection. This further corroborates the notion that NfL levels are independent of disease accumulation. Previous studies, however, have reported conflicting results. CSF NfL was reported to increase with increasing number of regions affected by both UMN and LMN degeneration (Poesen et al., 2017); several studies also showed that NfL correlated with UMN burden (defined clinically or by neuroimaging) but not with the extent of LMN damage (Menke et al., 2015; Gong et al., 2018; Schreiber et al., 2018). Conversely, a recent study identified a significant association of NfL with the number of regions affected by LMN degeneration, but not UMN damage (Abu-Rumeileh et al., 2020).

This study is not without limitations. Comprehensive genetic profiles were not available for the entire ALS cohort. Given that CSF NfL levels are reported to be higher in patients with *C9orf72* mutations (Huang et al., 2020) and lower in those with *SOD1* mutations (Zetterberg et al., 2007), this may also represent a confounding factor. Further studies are needed to clarify if genotype-specific differences exist independent of disease aggressiveness, as, for example, *C9orf72* expansion carriers are known to have a worse prognosis relative to patients with sporadic ALS or other familial mutations (Miltenberger-Miltenyi et al., 2019; Rooney et al., 2019). The presence of clinically overt FTD was assessed, but this should be examined in more detail in future studies, as previous data have indicated links between cognitive deterioration and NfL levels (Illán-Gala et al., 2018; Delaby et al., 2020). Furthermore, this study is limited to the analysis of NfL concentrations in the CSF. Owing to recent technical advances, assessment of serum NfL is becoming increasingly available and holds promise as a prognostic biomarker for ALS (Benatar et al., 2020). However, future large-scale studies with matched assessments in both serum and CSF are necessary to adequately compare the prognostic potential of NfL in both biofluids. While serum and CSF levels of NfL are known to correlate well (Gille et al., 2019; Benatar et al., 2020), the considerably less invasive manner of collection speaks in favor of using blood biomarkers. However, taking into consideration the proximity of CSF to the key pathological processes in ALS, we posit that CSF analyses should still play an important role in future research, as relevance has been demonstrated in this and other studies. Furthermore, a baseline lumbar puncture constitutes an essential step in the diagnostic workup of any patient with (suspected) ALS. Future studies should also incorporate pNfH and multicenter data, in order to fully explore the biomarker potential of neurofilaments.

Given the number of pseudolongitudinal CSF NfL data points analyzed in this study, our findings provide strong evidence for the ability of CSF NfL to reflect the rate of neuroaxonal degeneration in ALS and its potential to serve as a biomarker in future clinical trials. We show that the D50 progression model is an easily applicable and precise tool for investigating associations between biomarkers and clinical parameters in a heterogeneous ALS cohort. We recommend the use of this model for future ALS biomarker studies.

## Data Availability Statement

The raw data supporting the conclusions of this article will be made available by the authors, without undue reservation.

## Ethics Statement

The studies involving human participants were reviewed and approved by the Jena University Hospital Ethics Committee. The patients/participants provided their written informed consent to participate in this study.

## Author Contributions

MD, RS, NG, and JG contributed to conception and design of the study. JG developed the D50 model and the database. JG, BS, MD, RS, NG, and KM performed the data curation. NG and KM conducted the laboratory analyses. MD and RS performed the statistical analysis. MD wrote the first draft of the manuscript. MD, NG, and RS wrote sections of the manuscript. JG and OW provided the funding acquisition. JG conducted the Project administration and supervision. All authors contributed to manuscript revision, read, and approved the submitted version.

## Conflict of Interest

The authors declare that the research was conducted in the absence of any commercial or financial relationships that could be construed as a potential conflict of interest.

## Abbreviations

ALS: amyotrophic lateral sclerosis
ALSFRS-R: Amyotrophic Lateral Sclerosis Functional Rating Scale-Revised
ANCOVA: analysis of covariance
CSF: cerebrospinal fluid
ELISA: enzyme-linked immunosorbent assay
FTD: frontotemporal dementia
LMN: lower motor neuron
MiToS: Milano–Torino Staging System
NfL: neurofilament light chain
pNfH: phosphorylated neurofilament heavy chain
rD50: relative D50
SD: standard deviation
UMN: upper motor neuron.

## References

[B1] Abu-Rumeileh S. Vacchiano V. Zenesini C. Polischi B. De Pasqua S. Fileccia E. (2020). Diagnostic-prognostic value and electrophysiological correlates of CSF biomarkers of neurodegeneration and neuroinflammation in amyotrophic lateral sclerosis. *J. Neurol.* 267 1699–1708. 10.1007/s00415-020-09761-z 32100123

[B2] Andres-Benito P. Dominguez R. Colomina M. J. Llorens F. Povedano M. Ferrer I. (2018). YKL40 in sporadic amyotrophic lateral sclerosis: cerebrospinal fluid levels as a prognosis marker of disease progression. *Aging* 10 2367–2382. 10.18632/aging.101551 30215603 PMC6188478

[B3] Bakker L. A. Schroder C. D. Tan H. H. G. Vugts S. Van Eijk R. P. A. Van Es M. A. (2020). Development and assessment of the inter-rater and intra-rater reproducibility of a self-administration version of the ALSFRS-R. *J. Neurol. Neurosurg. Psychiatry* 91 75–81. 10.1136/jnnp-2019-321138 31558653

[B4] Benatar M. Wuu J. Andersen P. M. Lombardi V. Malaspina A. (2018). Neurofilament light: a candidate biomarker of presymptomatic amyotrophic lateral sclerosis and phenoconversion. *Ann. Neurol.* 84 130–139. 10.1002/ana.25276 30014505 PMC11348288

[B5] Benatar M. Wuu J. Lombardi V. Jeromin A. Bowser R. Andersen P. M. (2019). Neurofilaments in pre-symptomatic ALS and the impact of genotype. *Amyotroph. Lateral. Scler Frontotemporal. Degener.* 20 538–548. 10.1080/21678421.2019.1646769 31432691 PMC6768722

[B6] Benatar M. Zhang L. Wang L. Granit V. Statland J. Barohn R. (2020). Validation of serum neurofilaments as prognostic and potential pharmacodynamic biomarkers for ALS. *Neurology* 95 e59–e69. 32385188 10.1212/WNL.0000000000009559 PMC7371380

[B7] Brooks B. R. Miller R. G. Swash M. Munsat T. L. (2000). El Escorial revisited: revised criteria for the diagnosis of amyotrophic lateral sclerosis. *Amyotroph. Lateral. Scler. Other. Motor. Neuron. Disord.* 1 293–299. 10.1080/146608200300079536 11464847

[B8] Chiò A. Calvo A. Moglia C. Mazzini L. Mora G. (2011). Phenotypic heterogeneity of amyotrophic lateral sclerosis: a population based study. *J. Neurol. Neurosurg. Psychiatry* 82 740–746. 21402743 10.1136/jnnp.2010.235952

[B9] Chio A. Hammond E. R. Mora G. Bonito V. Filippini G. (2015). Development and evaluation of a clinical staging system for amyotrophic lateral sclerosis. *J. Neurol. Neurosurg. Psychiatry* 86 38–44. 24336810 10.1136/jnnp-2013-306589

[B10] de Carvalho M. Dengler R. Eisen A. England J. D. Kaji R. Kimura J. (2008). Electrodiagnostic criteria for diagnosis of ALS. *Clin. Neurophysiol.* 119 497–503. 10.1016/j.clinph.2007.09.143 18164242

[B11] Delaby C. Alcolea D. Carmona-Iragui M. Illán-Gala I. Morenas-Rodríguez E. Barroeta I. (2020). Differential levels of Neurofilament Light protein in cerebrospinal fluid in patients with a wide range of neurodegenerative disorders. *Sci. Rep.* 10:9161. 10.1038/s41598-020-66090-x 32514050 PMC7280194

[B12] Gaiani A. Martinelli I. Bello L. Querin G. Puthenparampil M. Ruggero S. (2017). Diagnostic and prognostic biomarkers in amyotrophic lateral sclerosis: neurofilament light chain levels in definite subtypes of disease. *JAMA Neurol.* 74 525–532. 10.1001/jamaneurol.2016.5398 28264096 PMC5822207

[B13] Gille B. De Schaepdryver M. Goossens J. Dedeene L. De Vocht J. Oldoni E. (2019). Serum neurofilament light chain levels as a marker of upper motor neuron degeneration in patients with amyotrophic lateral sclerosis. *Neuropathol Appl. Neurobiol.* 45 291–304. 10.1111/nan.12511 29908069

[B14] Gong Z. Y. Lv G. P. Gao L. N. Lu Y. Guo J. Zang D. W. (2018). Neurofilament Subunit L Levels in the cerebrospinal fluid and serum of patients with amyotrophic lateral sclerosis. *Neurodegener. Dis.* 18 165–172. 10.1159/000488681 29898446

[B15] Gordon P. H. Cheng B. Salachas F. Pradat P. F. Bruneteau G. Corcia P. (2010). Progression in ALS is not linear but is curvilinear. *J. Neurol.* 257 1713–1717. 10.1007/s00415-010-5609-1 20532545

[B16] Gray E. Oeckl P. Amador M. D. M. Andreasson U. An J. Blennow K. (2020). A multi-center study of neurofilament assay reliability and inter-laboratory variability. *Amyotroph. Lateral. Scler. Frontotemporal. Degener.* 21 452–458. 10.1080/21678421.2020.1779300 32558597

[B17] Huang F. Zhu Y. Hsiao-Nakamoto J. Tang X. Dugas J. C. Moscovitch-Lopatin M. (2020). Longitudinal biomarkers in amyotrophic lateral sclerosis. *Ann. Clin. Transl. Neurol.* 7 1103–1116. 32515902 10.1002/acn3.51078 PMC7359115

[B18] Illán-Gala I. Alcolea D. Montal V. Dols-Icardo O. Munoz L. De Luna N. (2018). CSF sAPPβ, YKL-40, and NfL along the ALS-FTD spectrum. *Neurology* 91 e1619–e1628. 30291183 10.1212/WNL.0000000000006383

[B19] Julien J. P. (2001). Amyotrophic lateral sclerosis. unfolding the toxicity of the misfolded. *Cell* 104 581–591. 11239414 10.1016/s0092-8674(01)00244-6

[B20] Kasai T. Kojima Y. Ohmichi T. Tatebe H. Tsuji Y. Noto Y. I. (2019). Combined use of CSF NfL and CSF TDP-43 improves diagnostic performance in ALS. *Ann. Clin. Transl. Neurol.* 6 2489–2502. 10.1002/acn3.50943 31742901 PMC6917342

[B21] Khalil M. Teunissen C. E. Otto M. Piehl F. Sormani M. P. Gattringer T. (2018). Neurofilaments as biomarkers in neurological disorders. *Nat. Rev. Neurol.* 14 577–589. 30171200 10.1038/s41582-018-0058-z

[B22] Lu C. H. Macdonald-Wallis C. Gray E. Pearce N. Petzold A. Norgren N. (2015). Neurofilament light chain: a prognostic biomarker in amyotrophic lateral sclerosis. *Neurology* 84 2247–2257. 10.1212/wnl.0000000000001642 25934855 PMC4456658

[B23] Menke R. A. Gray E. Lu C. H. Kuhle J. Talbot K. Malaspina A. (2015). CSF neurofilament light chain reflects corticospinal tract degeneration in ALS. *Ann. Clin. Transl. Neurol.* 2 748–755. 10.1002/acn3.212 26273687 PMC4531057

[B24] Miller A. M. Rutkowska A. Bahl J. M. Herukka S. K. Koel-Simmelink M. J. Kruse N. (2016). Multicenter immunoassay validation of cerebrospinal fluid neurofilament light: a biomarker for neurodegeneration. *Bioanalysis* 8 2243–2254. 10.4155/bio-2016-0114 27684648

[B25] Miller T. Cudkowicz M. Shaw P. J. Andersen P. M. Atassi N. Bucelli R. C. (2020). Phase 1-2 trial of antisense oligonucleotide tofersen for SOD1 ALS. *N. Engl. J. Med.* 383 109–119. 10.1056/nejmoa2003715 32640130

[B26] Miltenberger-Miltenyi G. Conceicao V. A. Gromicho M. Pronto-Laborinho A. C. Pinto S. Andersen P. M. (2019). C9orf72 expansion is associated with accelerated decline of respiratory function and decreased survival in amyotrophic lateral sclerosis. *J. Neurol. Neurosurg. Psychiatry* 90 118–120. 10.1136/jnnp-2018-318032 29661924

[B27] Oeckl P. Jardel C. Salachas F. Lamari F. Andersen P. M. Bowser R. (2016). Multicenter validation of CSF neurofilaments as diagnostic biomarkers for ALS. *Amyotroph. Lateral. Scler. Frontotemporal. Degener.* 17 404–413. 27415180 10.3109/21678421.2016.1167913

[B28] Olsson B. Alberg L. Cullen N. C. Michael E. Wahlgren L. Kroksmark A. K. (2019). NFL is a marker of treatment response in children with SMA treated with nusinersen. *J. Neurol.* 266 2129–2136. 10.1007/s00415-019-09389-8 31123861 PMC6687695

[B29] Paganoni S. Macklin E. A. Lee A. Murphy A. Chang J. Zipf A. (2014). Diagnostic timelines and delays in diagnosing amyotrophic lateral sclerosis (ALS). *Amyotroph. Lateral. Scler. Frontotemporal. Degener.* 15 453–456. 10.3109/21678421.2014.903974 24981792 PMC4433003

[B30] Petrov D. Mansfield C. Moussy A. Hermine O. (2017). ALS clinical trials review: 20 years of failure. Are we any closer to registering a new treatment? *Front. Aging Neurosci.* 9:68. 10.3389/fnagi.2017.00068 28382000 PMC5360725

[B31] Petzold A. (2005). Neurofilament phosphoforms: surrogate markers for axonal injury, degeneration and loss. *J. Neurol. Sci.* 233 183–198. 10.1016/j.jns.2005.03.015 15896809

[B32] Petzold A. Altintas A. Andreoni L. Bartos A. Berthele A. Blankenstein M. A. (2010). Neurofilament ELISA validation. *J. Immunol. Methods* 352 23–31. 19857497 10.1016/j.jim.2009.09.014

[B33] Pijnenburg Y. A. Verwey N. A. Van Der Flier W. M. Scheltens P. Teunissen C. E. (2015). Discriminative and prognostic potential of cerebrospinal fluid phosphoTau/tau ratio and neurofilaments for frontotemporal dementia subtypes. *Alzheimers Dement.* 1 505–512. 10.1016/j.dadm.2015.11.001 27239528 PMC4879490

[B34] Poesen K. De Schaepdryver M. Stubendorff B. Gille B. Muckova P. Wendler S. (2017). Neurofilament markers for ALS correlate with extent of upper and lower motor neuron disease. *Neurology* 88 2302–2309. 10.1212/wnl.0000000000004029 28500227

[B35] Prell T. Gaur N. Steinbach R. Witte O. W. Grosskreutz J. (2020). Modelling disease course in amyotrophic lateral Sclerosis: pseudo-longitudinal insights from cross-sectional health-related quality of life data. *Health Qual. Life Outcomes* 18:117. 10.1186/s12955-020-01372-6 PMC7195704 32357946

[B36] Prell T. Stubendorff B. Le T. T. Gaur N. Tadic V. Rodiger A. (2019). Reaction to endoplasmic reticulum stress via ATF6 in amyotrophic lateral sclerosis deteriorates with aging. *Front. Aging Neurosci.* 11:5. 10.3389/fnagi.2019.00005 30740050 PMC6355670

[B37] Roche J. C. Rojas-Garcia R. Scott K. M. Scotton W. Ellis C. E. Burman R. (2012). A proposed staging system for amyotrophic lateral sclerosis. *Brain* 135 847–852. 22271664 10.1093/brain/awr351 PMC3286327

[B38] Rooney J. Murray D. Campion A. Moloney H. Tattersall R. Doherty M. (2019). The C9orf72 expansion is associated with accelerated respiratory function decline in a large Amyotrophic Lateral Sclerosis cohort. *HRB Open Res.* 2:23. 10.12688/hrbopenres.12940.1 32296747 PMC7140774

[B39] Rosengren L. E. Karlsson J. E. Karlsson J. O. Persson L. I. Wikkelsø C. (1996). Patients with amyotrophic lateral sclerosis and other neurodegenerative diseases have increased levels of neurofilament protein in CSF. *J. Neurochem.* 67 2013–2018. 10.1046/j.1471-4159.1996.67052013.x 8863508

[B40] Rossi D. Volanti P. Brambilla L. Colletti T. Spataro R. La Bella V. (2018). CSF neurofilament proteins as diagnostic and prognostic biomarkers for amyotrophic lateral sclerosis. *J. Neurol.* 265 510–521. 10.1007/s00415-017-8730-6 29322259

[B41] Scarafino A. D’errico E. Introna A. Fraddosio A. Distaso E. Tempesta I. (2018). Diagnostic and prognostic power of CSF Tau in amyotrophic lateral sclerosis. *J. Neurol.* 265 2353–2362. 10.1007/s00415-018-9008-3 30116940

[B42] Schreiber S. Spotorno N. Schreiber F. Acosta-Cabronero J. Kaufmann J. Machts J. (2018). Significance of CSF NfL and tau in ALS. *J. Neurol.* 265 2633–2645. 10.1007/s00415-018-9043-0 30187162

[B43] Shefner J. M. Al-Chalabi A. Baker M. R. Cui L. Y. De Carvalho M. Eisen A. (2020). A proposal for new diagnostic criteria for ALS. *Clin. Neurophysiol.* 131 1975–1978. 32387049 10.1016/j.clinph.2020.04.005

[B44] Skillbäck T. Mattsson N. Blennow K. Zetterberg H. (2017). Cerebrospinal fluid neurofilament light concentration in motor neuron disease and frontotemporal dementia predicts survival. *Amyotroph. Lateral. Scler. Frontotemporal. Degener.* 18 397–403. 10.1080/21678421.2017.1281962 28631955

[B45] Strong M. J. Grace G. M. Freedman M. Lomen-Hoerth C. Woolley S. Goldstein L. H. (2009). Consensus criteria for the diagnosis of frontotemporal cognitive and behavioural syndromes in amyotrophic lateral sclerosis. *Amyotroph. Lateral Scler.* 10, 131–146. 10.1080/17482960802654364 19462523

[B46] Strong M. J. Abrahams S. Goldstein L. H. Woolley S. Mclaughlin P. Snowden J. (2017). Amyotrophic lateral sclerosis – frontotemporal spectrum disorder (ALS-FTSD): revised diagnostic criteria. *Amyotroph. Lateral Scler. Frontotemporal Degener.* 18, 153–174. 10.1080/21678421.2016.1267768 28054827 PMC7409990

[B47] Steinacker P. Anderl-Straub S. Diehl-Schmid J. Semler E. Uttner I. Von Arnim C. A. F. (2018a). Serum neurofilament light chain in behavioral variant frontotemporal dementia. *Neurology* 91 e1390–e1401. 30209235 10.1212/WNL.0000000000006318

[B48] Steinacker P. Verde F. Fang L. Feneberg E. Oeckl P. Roeber S. (2018b). Chitotriosidase (CHIT1) is increased in microglia and macrophages in spinal cord of amyotrophic lateral sclerosis and cerebrospinal fluid levels correlate with disease severity and progression. *J. Neurol. Neurosurg. Psychiatry* 89 239–247. 10.1136/jnnp-2017-317138 29142138

[B49] Steinacker P. Feneberg E. Weishaupt J. Brettschneider J. Tumani H. Andersen P. M. (2016). Neurofilaments in the diagnosis of motoneuron diseases: a prospective study on 455 patients. *J. Neurol. Neurosurg. Psychiatry* 87 12–20. 26296871 10.1136/jnnp-2015-311387

[B50] Steinbach R. Gaur N. Roediger A. Mayer T. E. Witte O. W. Prell T. (2020). Disease aggressiveness signatures of amyotrophic lateral sclerosis in white matter tracts revealed by the D50 disease progression model. *Hum. Brain Mapp.* 42 737–752. 10.1002/hbm.25258 33103324 PMC7814763

[B51] Sun Q. Zhao X. Li S. Yang F. Wang H. Cui F. (2020). csf neurofilament light chain elevation predicts ALS severity and progression. *Front. Neurol.* 11:919. 10.3389/fneur.2020.00919 32982935 PMC7484044

[B52] Tortelli R. Ruggieri M. Cortese R. D’errico E. Capozzo R. Leo A. (2012). Elevated cerebrospinal fluid neurofilament light levels in patients with amyotrophic lateral sclerosis: a possible marker of disease severity and progression. *Eur. J. Neurol.* 19 1561–1567. 10.1111/j.1468-1331.2012.03777.x 22680408

[B53] Vågberg M. Norgren N. Dring A. Lindqvist T. Birgander R. Zetterberg H. (2015). Levels and age dependency of neurofilament light and glial fibrillary acidic protein in healthy individuals and their relation to the brain parenchymal fraction. *PLoS One* 10:e0135886. 10.1371/journal.pone.0135886 26317831 PMC4552591

[B54] Van Den Berg L. H. Sorenson E. Gronseth G. Macklin E. A. Andrews J. Baloh R. H. (2019). Revised airlie house consensus guidelines for design and implementation of ALS clinical trials. *Neurology* 92 e1610–e1623. 30850440 10.1212/WNL.0000000000007242 PMC6448453

[B55] Zetterberg H. Jacobsson J. Rosengren L. Blennow K. Andersen P. M. (2007). Cerebrospinal fluid neurofilament light levels in amyotrophic lateral sclerosis: impact of SOD1 genotype. *Eur. J. Neurol.* 14 1329–1333. 10.1111/j.1468-1331.2007.01972.x 17903209

