# Supplementary material for: Cerebrospinal Fluid Neurofilament Light Chain (NfL) Predicts Disease Aggressiveness in Amyotrophic Lateral Sclerosis: An Application of the D50 Disease Progression Model
Source: Front Neurosci. 2021 Apr 6;15:651651. doi: 10.3389/fnins.2021.651651 (PMC8056017; doi:10.3389/fnins.2021.651651)
Supplement: Supplementary file 1 [file Data_Sheet_1.docx]

**Supplementary Tables**

| **disease control (*n* = 56)** | | **ALS mimic (*n* = 11)** | | **non-neurological control (*n* = 15)** | |
| --- | --- | --- | --- | --- | --- |
| 4 | parkinsonism | 1 | degenerative spondylopathy with radiculopathy | 1 | rheumatologic disorder |
| 8 | atypical parkinsonian syndrome | 1 | multifocal motor neuropathy | 1 | somatoform vertigo |
| 1 | parkinson and frontotemporal dementia | 3 | polyneuropathy | 1 | gait disorder due to accident |
| 2 | frontotemporal dementia | 2 | spinal stenosis | 1 | obstructive sleep apnea |
| 2 | dementia | 1 | ependymoma | 9 | benign fasciculations |
| 3 | mild cognitive impairment | 1 | proximal myotonic myopathy (PROMM) | 1 | schizophrenia |
| 2 | hereditary spastic paraplegia | 1 | adrenoleukodystrophy | 1 | psychic fatigue syndrome |
| 3 | gait disorder | 1 | spinal and bulbar muscle atrophy/ Kennedy disease |  |  |
| 2 | stroke |  |  |  |  |
| 1 | transient global amnesia |  |  |  |  |
| 1 | leukoencephalopathy |  |  |  |  |
| 1 | migraine |  |  |  |  |
| 3 | pseudotumor cerebri |  |  |  |  |
| 8 | normal pressure hydrocephalus |  |  |  |  |
| 1 | obstructive hydrocephalus |  |  |  |  |
| 1 | muscle hypertonia of unknown origin |  |  |  |  |
| 2 | myopathy |  |  |  |  |
| 3 | muscle fatigue of unknown origin |  |  |  |  |
| 1 | diabetic amyotrophy |  |  |  |  |
| 2 | chronic inflammatory demyelinating polyneuropathy |  |  |  |  |
| 1 | pain and dysesthesia of lower limbs of unknown origin |  |  |  |  |
| 1 | dermatomyositis |  |  |  |  |
| 1 | spinal disc herniation |  |  |  |  |
| 1 | vitamin B12 deficiency |  |  |  |  |
| 1 | chronic pain syndrome |  |  |  |  |

Supplementary Table 1 diagnoses of patients included in the study.

|  | | *Disease aggressiveness* | | | *p* |
| --- | --- | --- | --- | --- | --- |
|  | | high (D50 < 20) | intermediate (20 ≤ D50 < 40) | low (D50 ≥ 40) |  |
| *n* | | 37 | 37 | 37 |  |
| *Neurofilament light chain measurements* | | | | | |
| NfL (pg/ml) | | 17180.10  (7906.10 – 24843.00) | 8959.67  (4529.00 – 12157.00) | 4511.00  (2717.22 – 8967.00) | <0.001* |
| laboratory:  Germany/ Belgium | | 25 / 12  (67.6% / 32.4%) | 24/13  (64.9% / 35.1%) | 26 / 11  (70.3% / 29.7%) | 0.939 |
| *demographics* | | | | | |
| age at lumbar puncture | | 64.46 ± 10.02 | 62.56 ± 10.22 | 62.85 ± 9.83 | 0.679 |
| male/female | | 21 / 16  (56.8% / 43.2%) | 22 / 15  (59.5% / 40.5%) | 22 / 15  (59.5% / 40.5%) | 0.964 |
| *D50 disease progression model parameters* | | | | | |
| D50 $ | | 13.62 (8.87 – 16.16) | 28.06 (22.62 – 30.42) | 62.52 (46.55 – 99.42) | <0.001* |
| rD50 | | 0.34 ± 0.14 | 0.27 ± 0.10 | 0.24 ± 0.13 | <0.001* |
| Phase | I (rD50 < 0.25) | 11 (29.7%) | 15 (40.5%) | 18 (48.6%) | 0.284 |
|  | II (0.25 ≤ rD50 < 0.5) | 23 (62.2%) | 20 (54.1%) | 19 (51.4%) |  |
|  | III/IV (rD50 ≥ 0.5) | 3 (8.1%) | 2 (5.4%) | 0 (0%) |  |
| *Traditional disease metrics* | | | | | |
| ALSFRS-R at lumbar puncture $ | | 36 (32 – 40.50) | 40 (37 – 42.50) | 42 (36 – 44.50) | 0.003* |
| disease progression rate $ | | 1.44 (0.99 – 2.37) | 0.60 (0.49 – 0.80) | 0.21 (0.15 – 0.33) | <0.001* |
| disease duration at lumbar puncture (months) $ | | 8 (5.5 - 10.5) | 15 (10.5 - 17.5) | 25 (16.5 - 45.5) | <0.001* |
| Kings‘  stage | I | 8 (21.6%) | 12 (32.4%) | 16 (43.2%) | 0.082 |
|  | II | 10 (27%) | 13 (35.1%) | 15 (40.5%) |  |
|  | III | 14 (37.8%) | 8 (21.6%) | 6 (16.2%) |  |
|  | IV a | 3 (8.1%) | 1 (2.7%) | 0 (0%) |  |
|  | IV b | 2 (5.4%) | 3 (8.1%) | 0 (0%) |  |
|  | V | 0 (0%) | 0 (0%) | 0 (0%) |  |
| MiToS  stage | 0 | 18 (48.6%) | 31 (83.8%) | 31 (83.8%) | <0.001* |
|  | I | 15 (40.5%) | 4 (10.8%) | 6 (16.2%) |  |
|  | II | 4 (10.8%) | 2 (5.4%) | 0 (0%) |  |
|  | III-V | 0 (0%) | 0 (0%) | 0 (0%) |  |
| ALS phenotype | classic | 17 (45.9%) | 21 (56.8%) | 22 (59.5%) | 0.279 |
|  | bulbar | 16 (43.2%) | 13 (35.1%) | 9 (24.3%) |  |
|  | pyramidal | 3 (8.1%) | 3 (8.1%) | 2 (5.4%) |  |
|  | respiratory | 1 (2.7%) | 0 (0%) | 0 (0%) |  |
|  | flail arm | 0 (0%) | 0 (0%) | 3 (8.1%) |  |
|  | flail leg | 0 (0%) | 0 (0%) | 0 (0%) |  |
|  | pure LMN | 0 (0%) | 0 (0%) | 1 (2.1%) |  |
| Revised El Escorial | Definitive | 8 (21.6%) | 2 (5.4%) | 0 (0%) | <0.001* |
|  | Probable | 20 (54.1%) | 22 (59.5%) | 13 (35.1%) |  |
|  | Laboratory supported probable | 7 (18.9%) | 12 (32.4%) | 14 (37.8%) |  |
|  | possible | 2 (5.4%) | 1 (2.7%) | 6 (16.2%) |  |
|  | suspected | 0 (0%) | 0 (0%) | 4 (10.8%) |  |
| presence of FTD:  yes / no | | 2 (5.4%) | 2 (5.4%) | 1 (2.7%) | 1.000 |
| Riluzole treatment:  yes/ no | | 36 (97.3%) | 36 (97.3%) | 35 (94.6%) | 0.912 |

Supplementary Table 2 demographic and clinical data for patients with ALS in the filtered cohort for the ANCOVA (*n* = 111). Continuous variables with normal distribution are expressed as mean with standard deviation, non-parametric nominal variables are marked with $ and represented as median and interquartile range. Categorical variables are expressed as number and percentage. For the comparison of demographic and clinical variables amongst the three aggressiveness subgroups, analyses of covariance, Kruskal Wallis tests, chi square tests or Fisher-Freeman-Halton Exact tests were applied where appropriate. Asterisks * mark statistical significance at *p* < 0.05. *Abbreviations*: *ALS:* Amyotrophic Lateral Sclerosis, *ALSFRS-R:* revised ALS functional rating scale, *FTD:* frontotemporal dementia, *LMN*: lower motor neuron, *MiToS:* Milano Torino staging system, *NfL*: Neurofilament Light chain, *rD50*: relative D50.

| Tests of Between-Subjects Effects | | | |
| --- | --- | --- | --- |
| Dependent Variable: Log[NfL] | | | |
| Factor | df | F | *p* |
| disease aggressiveness (D50 subgroups) | 2 | 23.050 | 0.000 |
| rD50-derived disease Phase | 1 | 0.156 | 0.693 |
| age at LP | 1 | 4.557 | 0.035 |
| Laboratory | 1 | 9.118 | 0.003 |
| gender | 1 | 0.210 | 0.648 |
| FTD | 1 | 3.534 | 0.063 |
| ALS Phenotype | 1 | 0.008 | 0.931 |

Supplementary Table 3 ANCOVA results of the filtered cohort

Abbreviations: *ALS:* Amyotrophic Lateral Sclerosis, *ANCOVA:* Analysis of covariance, *FTD:* frontotemporal dementia, *LP:* lumbar puncture, *NfL*: Neurofilament Light chain.

|  | | **disease aggressiveness** | | | | | | Association with CSF NfL (ANOVA) |
| --- | --- | --- | --- | --- | --- | --- | --- | --- |
|  |  | **high** | | **intermediate** | | **low** | |  |
|  |  | n | % | n | % | n | % |  |
| number of regions with UMN/LMN involvement (clinical) | 1 | 1 | 2.3 | 4 | 6.6 | 8 | 15.4 | *p* = 0.059 |
|  | 2 | 14 | 32.6 | 22 | 36.1 | 19 | 36.5 |  |
|  | 3 | 28 | 65.1 | 35 | 57.4 | 25 | 48.1 |  |
| number of regions with LMN involvement (clinical) | 1 | 5 | 11.6 | 12 | 19.7 | 15 | 28.8 | *p* = 0.210 |
|  | 2 | 17 | 39.5 | 20 | 32.8 | 20 | 38.5 |  |
|  | 3 | 21 | 48.8 | 29 | 47.5 | 17 | 32.7 |  |
| number of regions with UMN involvement (clinical) | 1 | 11 | 25.6 | 22 | 36.1 | 27 | 51.9 | *p* = 0.060 |
|  | 2 | 17 | 39.5 | 26 | 42.6 | 17 | 32.7 |  |
|  | 3 | 15 | 34.9 | 13 | 21.3 | 8 | 15.4 |  |
| number of regions with LMN involvement (clinical + EMG) | 1 | 1 | 2.3 | 3 | 4.9 | 6 | 11.5 | *p* = 0.519 |
|  | 2 | 6 | 14.0 | 14 | 23.0 | 16 | 30.8 |  |
|  | 3 | 36 | 83.7 | 44 | 72.1 | 30 | 57.7 |  |
| number of regions with UMN/LMN involvement (clinical+ EMG) | 1 | 0 | 0 | 0 | 0 | 1 | 1.9 | *p* = 0.249 |
|  | 2 | 6 | 14.0 | 11 | 18.0 | 16 | 30.8 |  |
|  | 3 | 37 | 86.0 | 50 | 82.0 | 35 | 67.3 |  |
| number of regions with LMN involvement (EMG) | 1 | 9 | 20.9 | 11 | 18.0 | 12 | 23.1 | *p* = 0.218 |
|  | 2 | 21 | 48.8 | 25 | 41.0 | 22 | 42.3 |  |
|  | 3 | 13 | 30.2 | 25 | 41.0 | 18 | 34.6 |  |

Supplementary Table 4 number of regions affected at the time of sampling

*Abbreviations: CSF:* cerebrospinal fluid, *EMG:* Electromyography, *LMN:* lower motor neuron, *NfL:* Neurofilament light chain, *UMN:* upper motor neuron.

**Supplementary Figures**
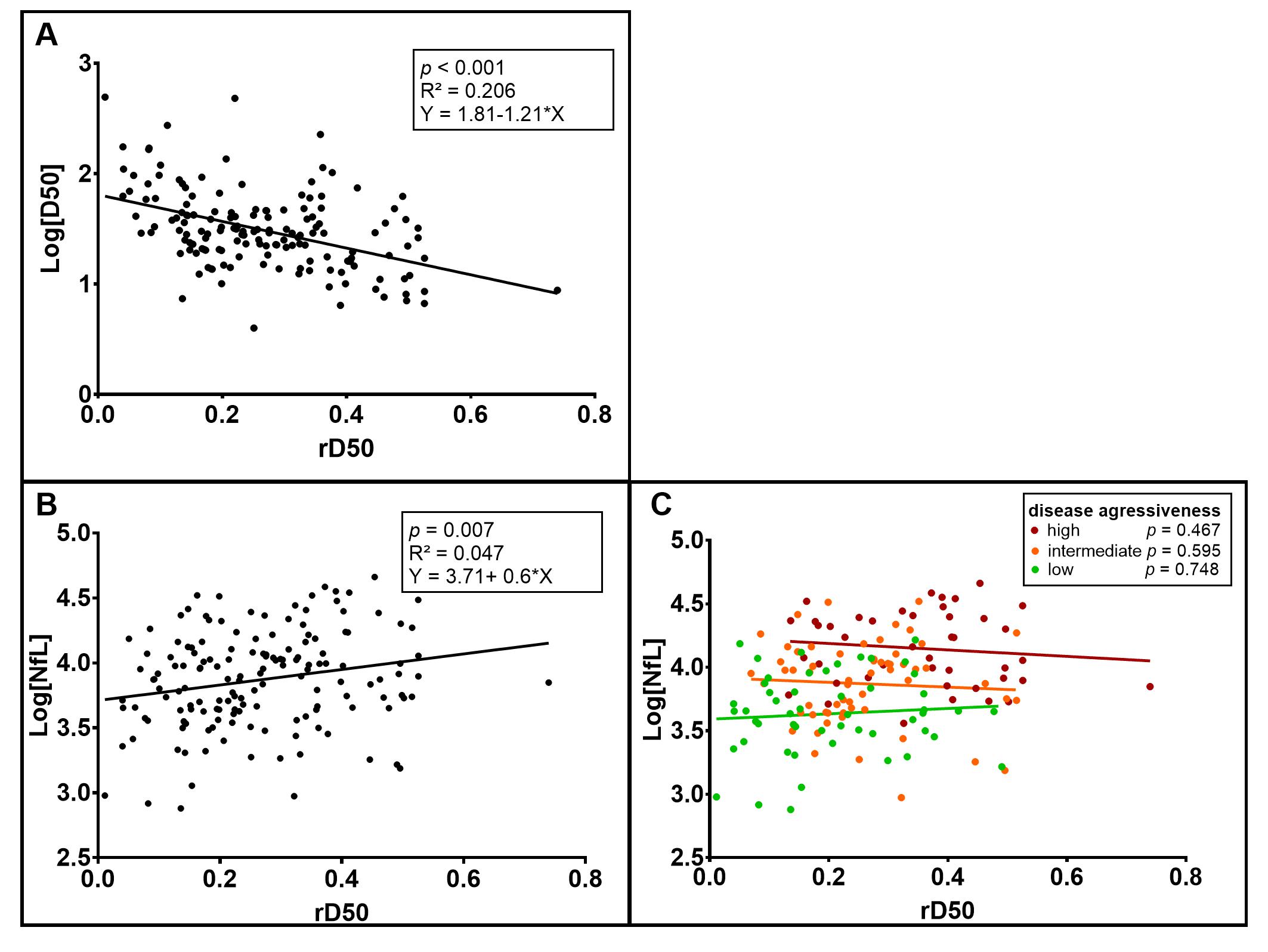


**Supplementary Figure 1** Sampling shift in aggressive disease causes apparent correlations of rD50 with NfL.

**(A)** Patients with low and intermediate disease aggressiveness were still in the earlier Phases of the disease at the time of sampling, while patients with highly aggressive disease had already reached later Phases by the time, they were referred to our center and lumbar puncture was performed. This is reflected by a negative correlation between rD50 and D50 in our ALS patient cohort (*p* < 0.001, ρ = -0.432). **(B)** For the entire ALS patient cohort, an apparent correlation between CSF NfL and rD50 could be calculated (*p* = 0.005, ρ = 0.224). **(C)** There was no significant correlation when stratifying patients into the three D50 subgroups (high in red: *p* = 0.467, intermediate in orange: *p* = 0.595, low disease aggressiveness in green: *p* = 0.748). This confirms that the aforementioned NfL-rD50 correlation (B) can be attributed to the sampling shift.

*Abbreviations*: *ALS:* Amyotrophic Lateral Sclerosis, *CSF:* cerebrospinal fluid, *NfL*: Neurofilament Light chain, *rD50*: relative D50.


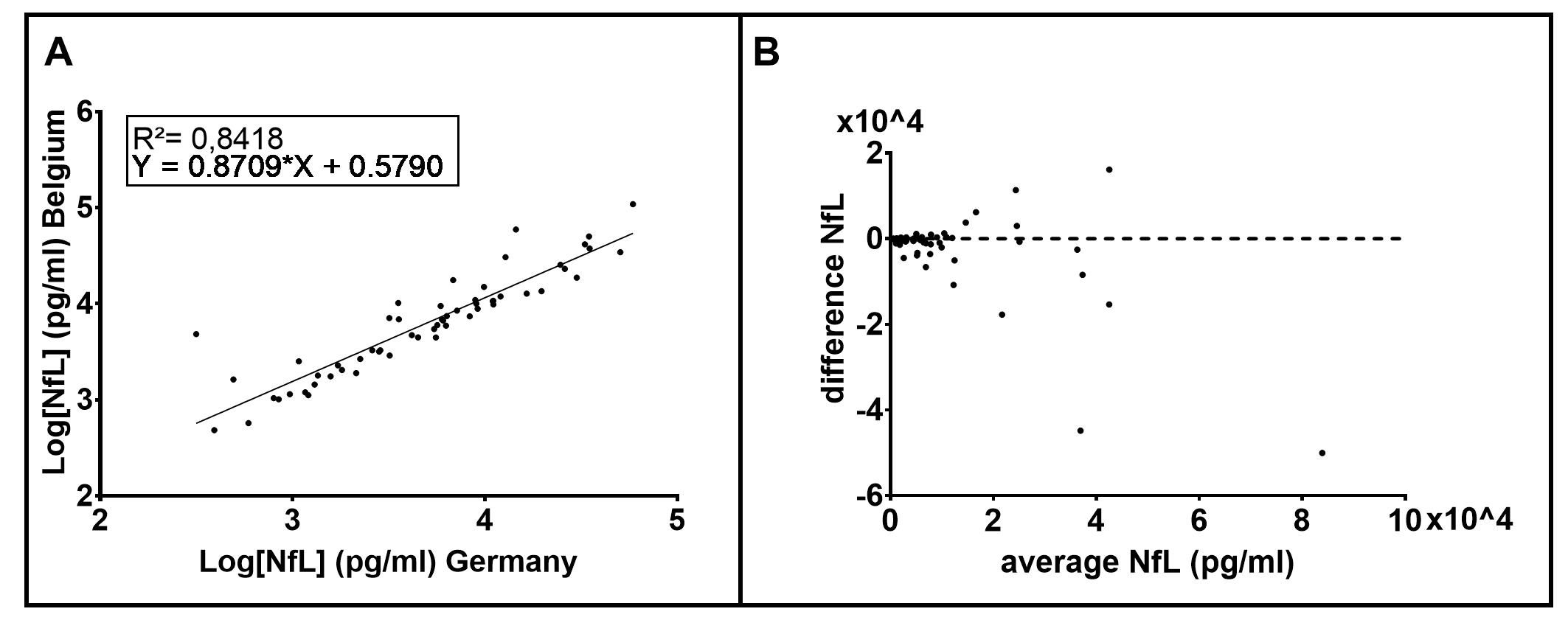


Supplementary Figure 2. Paired sample comparison of ELISA measurements in the two laboratories. (A) measurements from Germany and Belgium highly correlate. (B) Altman-Bland figure, indicating that CSF samples with higher NfL concentrations tended to have higher inter-laboratory variations. *Abbreviations*: *CSF:* Cerebrospinal fluid, *ELISA:* Enzyme linked immunosorbent assay*, NfL*: Neurofilament Light chain.
